# Long-term statin persistence is poor among high-risk patients with dyslipidemia: a real-world administrative claims analysis

**DOI:** 10.1186/s12944-019-1099-z

**Published:** 2019-09-16

**Authors:** Peter P. Toth, Craig Granowitz, Michael Hull, Amy Anderson, Sephy Philip

**Affiliations:** 1 CGH Medical Center, 101 East Miller Road, Sterling, IL 61081 USA; 2 Johns Hopkins University School of Medicine, Baltimore, MD USA; 3 Amarin Pharma Inc, Bedminster, NJ USA; 4 Optum, Eden Prairie, MN USA

**Keywords:** Statin, Triglycerides, Persistence, Discontinuation, Atherosclerotic cardiovascular disease, Diabetes

## Abstract

**Background:**

A decade ago, statin persistence was < 50% after 1 year, and recent short-term analyses have revealed very little progress in improving statin persistence, even in patients with a prior cardiovascular (CV) event. Data on longer-term statin persistence are lacking. We measured long-term statin persistence in patients with high CV risk.

**Methods:**

This retrospective administrative claims analysis of the Optum Research Database included patients aged ≥ 45 years with diabetes and/or atherosclerotic CV disease (ASCVD) who had a statin prescription filled in 2010. It included an elevated triglycerides (TG) cohort of patients with index date in 2010 and TG ≥ 150 mg/dL (*n* = 23,181) and a propensity-matched comparator cohort with TG < 150 mg/dL and high-density lipoprotein cholesterol > 40 mg/dL (*n* = 23,181). Both cohorts were followed for ≥ 6 months up to March 2016.

**Results:**

The probability of remaining on a prescription fill for index statin therapy was 47% after 1 year and 19% after 5 years in both cohorts. Statin persistence was worse among women than men, and among younger versus older patients (*P* < 0.001 for all comparisons). After 5 years, the probability of remaining on a prescription fill for index statin was < 25% across all subgroups assessed including patients with and without baseline revascularization, heart failure, peripheral artery disease and renal disease. Similar results were observed in a subcohort analysis of patients with TG 200–499 mg/dL.

**Conclusions:**

Long-term statin persistence after 5 years is alarmingly low (< 25%) and is a public health concern.

## Introduction

Statin therapy forms the cornerstone of both primary and secondary prevention and treatment of atherosclerotic cardiovascular disease (ASCVD) [1]. However, adherence and persistence to statin therapy are low, and this has been shown to negatively impact clinical outcomes and residual cardiovascular (CV) risk [2, 3]. Nearly a decade ago, statin persistence was reported to be less than 50% after 1 year [4]. A more recent study found that the proportion of days covered with a statin after a median follow-up of 2.2 years was 76%, with 40.5% of patients having poor adherence after 2 years [5]. Furthermore, adherence and persistence have been found to be low even in patients at high risk of CV events. In a recent study in which Medicare patients and patients with commercial and Medicare supplemental insurance were followed retrospectively for statin persistence, only 63.8% who started a statin following a myocardial infarction and < 40% of those with diabetes mellitus and a history of coronary heart disease and those without a history of coronary heart disease or diabetes mellitus took the medication with a high degree of adherence [6]. Another recent study in a Veterans Affairs population found an overall high adherence rate among patients taking a stable statin dose for secondary prevention of ASCVD of 87.7%; importantly, this study demonstrated a relationship between adherence and all-cause mortality [7].

Interpretation of results from these studies, however, has been complicated by the wide variability in estimates of statin persistence, as demonstrated in a recent systematic review [8]. This review found that statin persistence in primary prevention in the general population ranged from 7 to 84%, while persistence in secondary prevention for patients with a history of CV events ranged from 11.6 to 76.1%. Data on persistence with long-term statin use are lacking. Indeed, all but two of the studies in the systematic review were less than 3 years in duration; the longest study had a median follow-up of 4.1 years. Persistence in one of these studies was as low as 36.8% in secondary prevention and 23.3% in primary prevention [9]. Statin use among patients with diabetes is also low. In a recent study in patients with diabetes prescribed a statin, the mean proportion of days covered decreased from 0.69 at 6 months to 0.56 at 9 years, with the proportion considered adherent (proportion of days covered ≥ 0.80) decreasing from 54% at 6 months to 30.7% at 9 years [10].

Patients with elevated and high triglycerides (TG) and elevated low-density lipoprotein cholesterol (LDL-C) are at increased risk of CV events, and some residual CV risk remains even in those controlled on a statin [11, 12]. Statin adherence and persistence is therefore of particular interest in this high-risk group. The purpose of this study was to analyze long-term, real-world data on statin persistence in patients with elevated (≥ 150 mg/dL) and high (200–499 mg/dL) TG and high risk of CV disease, including those with diabetes and/or a history of ASCVD.

## Methods

### Study design

This was an observational retrospective administrative analysis of the Optum Research Database as previously described [13, 14]. The Optum Research Database is a claims database of > 160 million individuals with electronic health records for > 80 million individuals. The follow-up period, which was > 6 months, began on the index date and ended on the earliest of any of the following: the date of disenrollment from the plan, the date of death, or the end of the study on March 31, 2016. No patient identities or medical records were disclosed for the purposes of this study, and it was fully compliant with the Health Insurance Portability and Accountability Act. Measurement of index statin persistence was a secondary objective of the study.

The primary endpoint (frequency of major CV events in the follow-up period), and secondary endpoints (direct health care costs and resource utilization in the follow-up period) have been reported elsewhere [13, 14]. Other secondary prespecified analyses included statin persistence, as reported here.

### Study populations

Key inclusion criteria included: men and women aged ≥ 45 years on the index date; at least one prescription claim for statin therapy between January 1, 2010 and December 31, 2010, and ≥  6 months of baseline data prior to the index date (date of first statin claim); ≥ 1 medical claim with diagnosis code representing diabetes and/or ASCVD (ASCVD included acute coronary syndrome, myocardial infarction, angina, coronary or other arterial revascularization, stroke, transient ischemic attack, or peripheral artery disease [PAD]); and continuous enrollment with medical and pharmacy benefits during the baseline period and ≥  6 months starting on the index date, or death within 6 months of the index date. Key exclusion criteria included: niacin on the index date from a recent prescription fill, and ICD-9 codes indicating the presence of pregnancy, severe liver disease, acute or chronic pancreatitis, malabsorption syndrome, bypass surgery, HIV/AIDs, end-stage renal disease, hemodialysis, peritoneal dialysis, myositis, polymyositis, rhabdomyolysis, or drug or alcohol abuse.

Patients in an elevated-TG analysis cohort were required to have TG ≥ 150 mg/dL, while those in the comparator cohort were required to have TG < 150 mg/dL and high-density lipoprotein cholesterol (HDL-C) > 40 mg/dL [14]. In addition, a high-TG analysis subcohort (and corresponding comparator cohort) was investigated in patients with TG 200–499 mg/dL [13]. Concomitant use of ezetimibe, fibrates, and prescription omega-3 products was permitted. Data on fish oil dietary supplements were not captured in the claims database as they are not prescription products that generate claims.

### Statistical analysis

Persistence with index statin therapy as a class was measured as months to therapy discontinuation, inclusive of prescription fills on the index date. Patients who switched to a different type of statin were captured as persisting on statin therapy by this variable definition and were not captured as discontinuing. Persistence calculations were corrected for inpatient events under the assumption that medication would be supplied by the facility during the stay. Statin therapy was characterized as low, moderate, or high intensity. Ezetimibe was summarized together with statins, either as a low-intensity monotherapy or in combination with atorvastatin or simvastatin. Discontinuation from the index statin was defined as a gap in therapy of 30 days from the run-out date of days’ supply. Discontinuation was calculated within the first 6 months of the follow-up period, as well as for the duration of the follow-up period.

Persistence was calculated using descriptive statistics and with Kaplan-Meier probabilities. Persistence in different risk groups within the elevated- and high-TG and comparator cohorts was also calculated. These risk groups included gender, age, diabetes at baseline, ASCVD at baseline, and other CV diagnoses at baseline, including heart failure, PAD, renal disease, and a history of revascularization. Between-group comparisons were calculated as clustered *P* values using Cox proportional hazard models with cohort as an independent variable. A *P* value < 0.05 was considered statistically significant.

A propensity score analysis was used to create a matched comparator study cohort similar to the analysis cohort, but without elevated or high TG, by controlling for confounding relationships. A propensity score is a method of balancing cohorts and assumes that the distribution of observed baseline covariates is similar between the elevated-TG cohort and the comparator cohort. The estimated propensity score is the predicted probability of treatment derived from a fitted logistic regression model in which the cohort indicator is regressed on predetermined baseline characteristics. The method results in matched sets of patients from the two cohorts.

Propensity score matching was performed using a greedy match algorithm [15]. The procedure used attempts to match each case to a single control based on the first 8 digits of the propensity score, which was estimated using logistic regression, then 7 digits, etc., until a match was found. The closest available match, known as the nearest neighbor, was used. Ties were resolved randomly. A maximum allowed propensity score difference (ie, a caliper) of 0.01 between the matched case-control pairs was imposed a priori. Once a match was found, it was not reconsidered and the control was removed from the available pool for matches. The final sample of cases that were successfully matched to the controls was retained for analysis. The final list of variables included in the propensity score model was determined following review of the pre-matching descriptive analyses of patient characteristics and other pre-index measures and included age; gender; insurance type; region; baseline medical cost; LDL-C level relative to the median, if available; baseline use of statins, fibrates, or omega-3 fatty acids; and the following diagnoses: ASCVD, diabetes, stroke, hypertension, renal disease, and peripheral artery disease. Patients in the elevated-TG cohort were matched in a 1:1 ratio to the comparator cohort. Those who were not matched were not included in the descriptive analyses.

## Results

### Patients

Approximately 1.6 million patients with ≥ 1 prescription claim for a statin were identified from the Optum Research Database. A total of 23,181 propensity score–matched patients were included in the elevated-TG cohort (TG ≥ 150 mg/dL) with 23,181 corresponding patients in the comparator cohort (TG < 150 mg/dL and HDL-C > 40 mg/dL). As previously described, there were few clinically important differences between the elevated-TG and comparator cohorts, except for statistically significant differences in baseline lipids per the inclusion criteria due to the propensity score design (Table 1) [14]. The mean (SD) age was 62.2 (9.6) years and 62.6 (9.9) years in the elevated-TG and comparator cohorts, respectively; approximately 50% were women in both cohorts. Mean duration of follow-up was 41.4 and 42.5 months in the elevated-TG and comparator cohorts, respectively.

**Table 1:** Patient demographics, characteristics, and baseline comorbidities [14]

	Elevated-TG Cohort^a^ (*n* = 23,181)	Comparator Cohort^a^ (*n* = 23,181)	*P* Value
Age, mean (SD), years	62.2 (9.6)	62.6 (9.9)	<0.001
Female, *n* (%)	11,518 (49.7)	11,467 (49.5)	0.244
Insurance type, *n* (%)			
Commercial	15,823 (68.3)	15,855 (68.4)	0.461
Medicare	7358 (31.7)	7326 (31.6)	0.461
Duration of follow-up, mean (SD), months	41.4 (23.7)	42.5 (23.9)	<0.001
Baseline^b^ lipid profile, mean (SD), mg/dL			
TG	220.31 (77.4)	97.9 (28.9)	<0.001
LDL-C	104.6 (41.1)	100.9 (35.0)	<0.001
HDL-C	42.3 (10.2)	55.1 (12.2)	<0.001
Total cholesterol	190.2 (46.6)	175.4 (38.8)	<0.001
Non-HDL-C^c^	147.9 (44.2)	120.4 (36.5)	<0.001
Baseline comorbidities, *n* (%)			
Diabetes	19,392 (83.7)	19,478 (84.0)	0.017
ASCVD	6915 (29.8)	6800 (29.3)	0.009
MI	495 (2.1)	411 (1.8)	0.003
Stroke	750 (3.2)	674 (2.9)	0.005
Angina	1225 (5.3)	1179 (5.1)	0.284
Coronary revascularization	600 (2.6)	506 (2.2)	0.002
Peripheral artery disease	3384 (14.6)	3317 (14.3)	0.104
Heart failure	1258 (5.4)	1088 (4.7)	<0.001
Atrial fibrillation	1133 (4.9)	989 (4.3)	0.001
Hypertension	18,346 (79.1)	18,375 (79.3)	0.462
Renal disease	2832 (12.2)	2782 (12.0)	0.196

Rao-Scott test was used for binary measures. Robust standard errors were used for continuous measures

^a^Elevated TG ≥150 mg/dL and matched comparator with TG < 150 mg/dL and HDL-C > 40 mg/dL

^b^Baseline period excludes index date

^c^Calculated by subtracting HDL-C result from total cholesterol. This value was not calculated unless patients had both HDL-C and total cholesterol laboratory result in period

*ASCVD* atherosclerotic cardiovascular disease, *HDL-C* high-density lipoprotein cholesterol, *LDL-C* low-density lipoprotein cholesterol, *MI* myocardial infarction, *non-HDL-C* non-high-density lipoprotein cholesterol, *SD* standard deviation, *TG* triglycerides

Most baseline comorbidities were similar in the elevated-TG and comparator cohorts [14]. Consistent with the study entry criteria requiring a diagnosis of diabetes or ASCVD, 84% of patients in both cohorts had diabetes and 30 and 29% had ASCVD in the elevated-TG cohort and comparator cohort, respectively; in addition, 79% had hypertension in both cohorts. With the exception of PAD (14–15%) and renal disease (12%), all other comorbid diagnoses (myocardial infarction, stroke, angina, coronary revascularization, heart failure, atrial fibrillation, and transient ischemic attack) were present in < 10% of patients in both cohorts.

In addition to statins, during the first 6 months, 7% of patients in both the elevated-TG cohort and comparator cohort were prescribed fibrates, 8% were prescribed ezetimibe, and 2% received prescriptions for omega-3 fatty acids.

A parallel analysis in a subcohort of patients with high TG (200–499 mg/dL; *n* = 10,990) and a propensity-matched comparator group (TG < 150 mg/dL and HDL-C > 40 mg/dL; n = 10,990) was also conducted with similar demographic and baseline characteristic results [13].

### Statin persistence

**Fig. 1:**
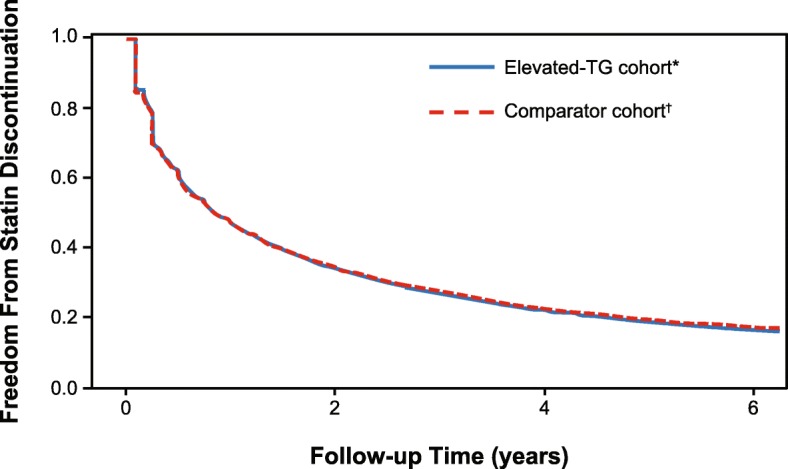
Kaplan-Meier Estimate of Persistence to Index Statin Therapy by Patients With High CV Risk. *TG ≥150 mg/dL. ^†^TG < 150 mg/dL and HDL-C > 40 mg/dL; propensity score matched to elevated-TG cohort. CV, cardiovascular; HDL-C, high-density lipoprotein cholesterol; TG, triglycerides

The proportion of days covered (ie, the proportion of days on which patients had index statin available) is shown in Table 2. For those patients who discontinued index statin therapy, the mean (SD) time to discontinuation was approximately 10.4 months and 10.3 months in the elevated-TG and comparator cohorts, respectively (Table 2). Among patients who discontinued index statin therapy, 55.6% in the elevated-TG cohort and 56.7% in the comparator cohort did so within the first 6 months (*P* = 0.036 for comparison). Kaplan-Meier estimates of the time to discontinuation are shown in Fig. 1. After 1 year, the probability of remaining on a prescription fill for index statin was 47% in both the elevated-TG cohort and its comparator cohort. At 5 years, the probability of these patients remaining on a prescription fill for index statin therapy fell to 19%, with no significant difference between the two cohorts (clustered *P* value for elevated-TG cohort vs comparator cohort, 0.511).

**Table 2:** Patient persistence to index statin therapy

Persistence Parameter, Mean (SD)	Elevated-TG Cohort^a^ (*n* = 23,181)	Comparator Cohort^b^ (*n* = 23,181)	*P* Value
6-month PDC	0.77 (0.26)	0.77 (0.26)	0.179
Overall PDC	0.68 (0.29)	0.68 (0.29)	0.147
Months to discontinuation^c^	10.4 (13.1)	10.3 (13.1)	0.599

^a^TG ≥150 mg/dL

^b^TG < 150 mg/dL and HDL-C > 40 mg/dL; propensity score matched to elevated-TG cohort

^c^For patients who discontinued

Rao-Scott test was used for binary measures; robust standard errors were used for continuous measures

*P* values calculated for comparison between the elevated-TG cohort and its propensity-matched comparator cohort

*HDL-C* high-density lipoprotein cholesterol, *PDC* proportion of days covered, *SD* standard deviation, *TG* triglycerides

In the parallel analysis of the subcohort of patients with high TG (TG > 200–499 mg/dL) versus a propensity-matched comparator group (TG < 150 mg/dL and HDL-C > 40 mg/dL), similar results were observed overall and in all subgroups tested below. These results are summarized in Tables 3, 4, 5, 6, 7, 8, 9, 10 and 11.

**Table 3:** Persistence to index statin therapy by patients from the high TG (200–499 mg/dL) and matched comparator cohorts

Persistence Parameter, Mean (SD)	High-TG Subcohort^a^ (*n* = 10,990)	Comparator Cohort^b^ (n = 10,990)	*P* Value
6-month PDC	0.76 (0.26)	0.76 (0.26)	0.707
Overall PDC	0.67 (0.30)	0.68 (0.29)	0.012
Months to discontinuation^c^	10.1 (12.7)	9.9 (12.7)	0.284

^a^TG ≥200–499 mg/dL

^b^TG < 150 mg/dL and HDL-C > 40 mg/dL; propensity score matched to high-TG cohort

^c^For patients who discontinued

Rao-Scott test was used for binary measures; robust standard errors were used for continuous measures

*P* values calculated for comparison between the high-TG cohort and its propensity-matched comparator cohort

*HDL-C* high-density lipoprotein cholesterol, *PDC* proportion of days covered, *SD* standard deviation, *TG* triglycerides

**Table 4:** Statin persistence over time according to gender

Cohort		Time (Years)					
		0.5	1	2	3	4	5
A) Elevated-TG analysis							
Study population: male (1)	Proportion	0.6278	0.4957	0.3625	0.2951	0.2459	0.2134
	At risk	7293	4879	2765	1847	1092	733
Study population: female (2)	Proportion	0.5819	0.4462	0.3150	0.2412	0.1962	0.1634
	At risk	6666	4393	2498	1601	942	638
Comparison group: male (3)	Proportion	0.6281	0.5070	0.3777	0.3047	0.2535	0.2175
	At risk	7319	5076	2987	2002	1177	790
Comparison group: female (4)	Proportion	0.5674	0.4367	0.3101	0.2450	0.1995	0.1694
	At risk	6477	4320	2427	1635	941	645
Clustered *P* values for between-group comparisons in the elevated-TG analysis							
		1 vs 2	1 vs 3	1 vs 4	2 vs 3	2 vs 4	3 vs 4
		<0.001	0.072	<0.001	<0.001	0.290	<0.001
B) High-TG analysis							
Study population: male (1)	Proportion	0.6078	0.4756	0.3453	0.2766	0.2245	0.1944
	At risk	3365	2217	1257	819	485	325
Study population: female (2)	Proportion	0.5738	0.4369	0.3018	0.2321	0.1842	0.1534
	At risk	3100	2024	1114	726	414	280
Comparison group: male (3)	Proportion	0.6133	0.4945	0.3641	0.2938	0.2543	0.2216
	At risk	3399	2349	1358	910	557	371
Comparison group: female (4)	Proportion	0.5607	0.4240	0.3012	0.2401	0.1918	0.1632
	At risk	3029	1979	1116	747	416	279
Clustered *P* values for between-group comparisons in the high-TG analysis							
		1 vs 2	1 vs 3	1 vs 4	2 vs 3	2 vs 4	3 vs 4
		<0.001	0.007	<0.001	<0.001	0.527	<0.001

Kaplan-Meier analysis. Clustered *P* values were calculated using Cox proportional hazard model with cohort as independent variable. Study population is the elevated-TG cohort (TG ≥150 mg/dL) and high-TG subcohort and their propensity score matched comparators with TG < 150 mg/dL and HDL-C > 40 mg/dL

*HDL-C* high-density lipoprotein cholesterol, *TG* triglycerides

**Table 5:** Statin persistence over time according to age strata at baseline

Cohort		Time (Years)							
		0.5	1	2	3	4	5		
A) Elevated-TG analysis									
Study population with age category 45–54 (1)	Proportion	0.5244	0.3851	0.2615	0.2024	0.1643	0.1353		
	At risk	2819	1770	936	603	363	219		
Study population with age category 55–64 (2)	Proportion	0.6025	0.4673	0.3377	0.2636	0.2118	0.1794		
	At risk	5661	3756	2028	1263	700	443		
Study population with age category 65+ (3)	Proportion	0.6596	0.5308	0.3898	0.3144	0.2655	0.2295		
	At risk	5479	3746	2299	1582	971	709		
Comparison group with age category 45–54 (4)	Proportion	0.5011	0.3824	0.2674	0.2028	0.1559	0.1310		
	At risk	2678	1756	971	624	341	237		
Comparison group with age category 55–64 (5)	Proportion	0.6108	0.4803	0.3496	0.2814	0.2317	0.1982		
	At risk	5708	3901	2135	1350	761	492		
Comparison group with age category 65+ (6)	Proportion	0.6454	0.5201	0.3867	0.3137	0.2651	0.2274		
	At risk	5410	3739	2308	1663	1016	706		
Clustered *P* values for between-group comparisons in the elevated-TG analysis									
		1 vs 2	1 vs 3	1 vs 4	1 vs 5	1 vs 6	2 vs 3	2 vs 4	2 vs 5
		<0.001	<0.001	0.494	<0.001	<0.001	<0.001	<0.001	0.016
		2 vs 6	3 vs 4	3 vs 5	3 vs 6	4 vs 5	4 vs 6	5 vs 6	
		<0.001	<0.001	<0.001	0.312	<0.001	<0.001	<0.001	
B) High-TG analysis									
Study population with age category 45–54 (1)	Proportion	0.5138	0.3693	0.2530	0.1883	0.1499	0.1243		
	At risk	1394	857	462	282	161	102		
Study population with age category 55–64 (2)	Proportion	0.5844	0.4492	0.3170	0.2521	0.1987	0.1686		
	At risk	2624	1726	914	590	325	204		
Study population with age category 65+ (3)	Proportion	0.6546	0.5281	0.3827	0.3048	0.2498	0.2143		
	At risk	2447	1658	995	673	413	299		
Comparison group with age category 45–54 (4)	Proportion	0.4846	0.3701	0.2648	0.2055	0.1620	0.1360		
	At risk	1300	850	484	315	176	117		
Comparison group with age category 55–64 (5)	Proportion	0.6024	0.4661	0.3322	0.2686	0.2249	0.1965		
	At risk	2679	1794	970	611	347	221		
Comparison group with age category 65+ (6)	Proportion	0.6416	0.5148	0.3816	0.3089	0.2638	0.2280		
	At risk	2449	1684	1020	731	450	312		
Clustered *P* values for between-group comparisons in the high-TG analysis									
		1 vs 2	1 vs 3	1 vs 4	1 vs 5	1 vs 6	2 vs 3	2 vs 4	2 vs 5
		<0.001	<0.001	0.849	<0.001	<0.001	<0.001	<0.001	0.019
		2 vs 6	3 vs 4	3 vs 5	3 vs 6	4 vs 5	4 vs 6	5 vs 6	
		<0.001	<0.001	<0.001	0.838	<0.001	<0.001	<0.001	

Kaplan-Meier analysis. Clustered *P* values were calculated using Cox proportional hazard model with cohort as independent variable. Study population is the elevated-TG cohort (TG ≥150 mg/dL) and high-TG subcohort and their propensity score matched comparators with TG < 150 mg/dL and HDL-C > 40 mg/dL

*HDL-C* high-density lipoprotein cholesterol, *TG* triglycerides

**Table 6:** Statin persistence over time in patient subgroup with ASCVD

Cohort		Time (Years)					
		0.5	1	2	3	4	5
A) Elevated-TG analysis							
Study population with baseline ASCVD (1)	Proportion	0.6200	0.4829	0.3537	0.2805	0.2372	0.2028
	At risk	4247	2830	1623	1053	640	439
Study population with no baseline ASCVD (2)	Proportion	0.5986	0.4661	0.3325	0.2628	0.2140	0.1819
	At risk	9712	6442	3640	2395	1394	932
Comparison group with baseline ASCVD (3)	Proportion	0.6171	0.4884	0.3560	0.2856	0.2350	0.2023
	At risk	4157	2830	1623	1098	626	427
Comparison group with no baseline ASCVD (4)	Proportion	0.5902	0.4655	0.3392	0.2707	0.2232	0.1900
	At risk	9639	6566	3791	2539	1492	1008
Clustered *P* values for between-group comparisons in the elevated-TG analysis							
		1 vs 2	1 vs 3	1 vs 4	2 vs 3	2 vs 4	3 vs 4
		0.001	0.801	0.005	<0.001	0.520	0.002
B) High-TG analysis							
Study population with baseline ASCVD (1)	Proportion	0.6193	0.4722	0.3411	0.2691	0.2228	0.1949
	At risk	1952	1247	710	464	277	198
Study population with no baseline ASCVD (2)	Proportion	0.5795	0.4500	0.3166	0.2485	0.1970	0.1653
	At risk	4513	2994	1661	1081	622	407
Comparison group with baseline ASCVD (3)	Proportion	0.6033	0.4778	0.3507	0.2754	0.2279	0.1932
	At risk	1877	1271	724	476	263	174
Comparison group with no baseline ASCVD (4)	Proportion	0.5809	0.4524	0.3260	0.2640	0.2214	0.1922
	At risk	4551	3057	1750	1181	710	476
Clustered *P* values for between-group comparisons in the high-TG analysis							
		1 vs 2	1 vs 3	1 vs 4	2 vs 3	2 vs 4	3 vs 4
		0.001	0.943	0.066	0.001	0.063	0.058

Kaplan-Meier analysis. Clustered *P* values were calculated using Cox proportional hazard model with cohort as independent variable. Study population is the elevated-TG cohort (TG ≥150 mg/dL) and high-TG subcohort and their propensity score matched comparators with TG < 150 mg/dL and HDL-C > 40 mg/dL

*ASCVD* atherosclerotic cardiovascular disease, *HDL-C* high-density lipoprotein cholesterol, *TG* triglycerides

**Table 7:** Statin persistence over time in patient subgroup with diabetes

Cohort		Time (Years)					
		0.5	1	2	3	4	5
A) Elevated-TG analysis							
Study population with baseline diabetes (1)	Proportion	0.6028	0.4692	0.3369	0.2653	0.2173	0.1840
	At risk	11,644	7716	4369	2851	1663	1113
Study population with no baseline diabetes (2)	Proportion	0.6163	0.4811	0.3487	0.2821	0.2391	0.2085
	At risk	2315	1556	894	597	371	258
Comparison group with baseline diabetes (3)	Proportion	0.5950	0.4684	0.3405	0.2710	0.2236	0.1904
	At risk	11,537	7855	4519	3010	1762	1194
Comparison group with no baseline diabetes (4)	Proportion	0.6144	0.4920	0.3633	0.2966	0.2432	0.2105
	At risk	2259	1541	895	627	356	241
Clustered *P* values for between-group comparisons in the elevated-TG analysis							
		1 vs 2	1 vs 3	1 vs 4	2 vs 3	2 vs 4	3 vs 4
		0.029	0.723	0.001	0.045	0.376	0.002
B) High-TG analysis							
Study population with baseline diabetes (1)	Proportion	0.5881	0.4551	0.3232	0.2532	0.2021	0.1701
	At risk	5463	3600	2013	1309	754	503
Study population with no baseline diabetes (2)	Proportion	0.6074	0.4642	0.3262	0.2610	0.2171	0.1944
	At risk	1002	641	358	236	145	102
Comparison group with baseline diabetes (3)	Proportion	0.5845	0.4552	0.3281	0.2639	0.2211	0.1909
	At risk	5460	3671	2094	1403	838	563
Comparison group with no baseline diabetes (4)	Proportion	0.6036	0.4854	0.3619	0.2868	0.2351	0.2021
	At risk	968	657	380	254	135	87
Clustered *P* values for between-group comparisons in the high-TG analysis							
		1 vs 2	1 vs 3	1 vs 4	2 vs 3	2 vs 4	3 vs 4
		0.259	0.246	0.005	0.572	0.168	0.025

Kaplan-Meier analysis. Clustered *P* values were calculated using Cox proportional hazard model with cohort as independent variable. Study population is the elevated-TG cohort (TG ≥150 mg/dL) and high-TG subcohort and their propensity score matched comparators with TG < 150 mg/dL and HDL-C > 40 mg/dL

*HDL-C* high-density lipoprotein cholesterol, *TG* triglycerides

**Table 8:** Statin persistence over time in patient subgroup with revascularization

Cohort		Time (Years)					
		0.5	1	2	3	4	5
A) Elevated-TG analysis							
Study population with baseline revascularization (1)	Proportion	0.6620	0.5162	0.3687	0.3120	0.2424	0.2049
	At risk	393	257	136	88	40	21
Study population with no baseline revascularization (2)	Proportion	0.6035	0.4699	0.3380	0.2670	0.2203	0.1876
	At risk	13,566	9015	5127	3360	1994	1350
Comparison group with baseline revascularization (3)	Proportion	0.6697	0.5362	0.3777	0.3076	0.2570	0.2124
	At risk	336	229	122	85	52	35
Comparison group with no baseline revascularization (4)	Proportion	0.5965	0.4708	0.3434	0.2743	0.2260	0.1931
	At risk	13,460	9167	5292	3552	2066	1400
Clustered *P* values for between-group comparisons in the elevated-TG analysis							
		1 vs 2	1 vs 3	1 vs 4	2 vs 3	2 vs 4	3 vs 4
		0.051	0.336	0.062	0.002	0.580	0.003
B) High-TG analysis							
Study population with baseline revascularization (1)	Proportion	0.6526	0.5026	0.3474	0.2920	0.2177	0.1910
	At risk	191	120	61	42	19	9
Study population with no baseline revascularization (2)	Proportion	0.5893	0.4552	0.3230	0.2534	0.2040	0.1734
	At risk	6274	4121	2310	1503	880	596
Comparison group with baseline revascularization (3)	Proportion	0.6620	0.5094	0.3659	0.2928	0.2560	0.2058
	At risk	141	93	53	36	24	16
Comparison group with no baseline revascularization (4)	Proportion	0.5858	0.4587	0.3324	0.2668	0.2226	0.1923
	At risk	6287	4235	2421	1621	949	634
Clustered *P* values for between-group comparisons in the high-TG analysis							
		1 vs 2	1 vs 3	1 vs 4	2 vs 3	2 vs 4	3 vs 4
		0.153	0.483	0.263	0.039	0.124	0.075

Kaplan-Meier analysis. Clustered *P* values were calculated using Cox proportional hazard model with cohort as independent variable. Study population is the elevated-TG cohort (TG ≥150 mg/dL) and high-TG subcohort and their propensity score matched comparators with TG < 150 mg/dL and HDL-C > 40 mg/dL

*HDL-C* high-density lipoprotein cholesterol, *TG* triglycerides

**Table 9:** Statin persistence over time in patient subgroup with heart failure

Cohort		Time (Years)					
		0.5	1	2	3	4	5
A) Elevated-TG analysis							
Study population with baseline CHF (1)	Proportion	0.6407	0.4965	0.3838	0.2977	0.2467	0.2170
	At risk	781	498	297	185	109	78
Study population with no baseline CHF (2)	Proportion	0.6030	0.4697	0.3364	0.2664	0.2194	0.1865
	At risk	13,178	8774	4966	3263	1925	1293
Comparison group with baseline CHF (3)	Proportion	0.6286	0.4885	0.3642	0.2925	0.2474	0.2119
	At risk	667	435	248	160	92	64
Comparison group with no	Proportion	0.5966	0.4714	0.3432	0.2742	0.2257	0.1927
baseline CHF (4)	At risk	13,129	8961	5166	3477	2026	1371
Clustered *P* values for between-group comparisons in the elevated-TG analysis							
		1 vs 2	1 vs 3	1 vs 4	2 vs 3	2 vs 4	3 vs 4
		0.010	0.777	0.019	0.045	0.426	0.071
B) High-TG analysis							
Study population with baseline CHF (1)	Proportion	0.6275	0.4813	0.3888	0.3019	0.2440	0.2235
	At risk	378	235	147	89	50	39
Study population with no baseline CHF (2)	Proportion	0.5888	0.4550	0.3200	0.2517	0.2021	0.1711
	At risk	6087	4006	2224	1456	849	566
Comparison group with baseline CHF (3)	Proportion	0.5962	0.4514	0.3563	0.2788	0.2360	0.1929
	At risk	303	195	118	76	42	27
Comparison group with no baseline CHF (4)	Proportion	0.5869	0.4600	0.3319	0.2667	0.2227	0.1926
	At risk	6125	4133	2356	1581	931	623
Clustered *P* values for between-group comparisons in the high-TG analysis							
		1 vs 2	1 vs 3	1 vs 4	2 vs 3	2 vs 4	3 vs 4
		0.026	0.336	0.093	0.419	0.059	0.761

Kaplan-Meier analysis. Clustered *P* values were calculated using Cox proportional hazard model with cohort as independent variable. Study population is the elevated-TG cohort (TG ≥150 mg/dL) and high-TG subcohort and their propensity score matched comparators with TG < 150 mg/dL and HDL-C > 40 mg/dL

*CHF* congestive heart failure, *HDL-C* high-density lipoprotein cholesterol, *TG* triglycerides

**Table 10:** Statin persistence over time in patient subgroup with peripheral artery disease

Cohort		Time (Years)					
		0.5	1	2	3	4	5
A) Elevated-TG analysis							
Study population with baseline PAD (1)	Proportion	0.6327	0.4965	0.3681	0.2866	0.2444	0.2110
	At risk	2117	1427	835	547	341	233
Study population with no baseline PAD (2)	Proportion	0.6003	0.4668	0.3338	0.2649	0.2168	0.1841
	At risk	11,842	7845	4428	2901	1693	1138
Comparison group with baseline PAD (3)	Proportion	0.6182	0.4960	0.3653	0.2994	0.2493	0.2130
	At risk	2022	1412	825	578	331	229
Comparison group with no baseline PAD (4)	Proportion	0.5947	0.4682	0.3406	0.2710	0.2229	0.1903
	At risk	11,774	7984	4589	3059	1787	1206
Clustered *P* values for between-group comparisons in the elevated-TG analysis							
		1 vs 2	1 vs 3	1 vs 4	2 vs 3	2 vs 4	3 vs 4
		< 0.001	0.911	0.001	< 0.001	0.431	0.003
B) High-TG analysis							
Study population with baseline PAD (1)	Proportion	0.6279	0.4780	0.3465	0.2677	0.2216	0.1967
	At risk	966	622	364	236	144	105
Study population with no baseline PAD (2)	Proportion	0.5849	0.4529	0.3199	0.2522	0.2015	0.1699
	At risk	5499	3619	2007	1309	755	500
Comparison group with baseline PAD (3)	Proportion	0.6067	0.4925	0.3619	0.2921	0.2418	0.2066
	At risk	925	655	377	259	146	103
Comparison group with no baseline PAD (4)	Proportion	0.5841	0.4543	0.3283	0.2632	0.2203	0.1903
	At risk	5503	3673	2097	1398	827	547
Clustered *P* values for between-group comparisons in the high-TG analysis							
		1 vs 2	1 vs 3	1 vs 4	2 vs 3	2 vs 4	3 vs 4
		0.016	0.680	0.107	0.004	0.121	0.037

Kaplan-Meier analysis. Clustered *P* values were calculated using Cox proportional hazard model with cohort as independent variable. Study population is the elevated-TG cohort (TG ≥150 mg/dL) and high-TG subcohort and their propensity score matched comparators with TG < 150 mg/dL and HDL-C > 40 mg/dL

*HDL-C* high-density lipoprotein cholesterol, *PAD* peripheral artery disease, *TG* triglycerides

**Table 11:** Statin persistence over time in patient subgroup with renal disease

Cohort		Time (Years)					
		0.5	1	2	3	4	5
A) Elevated-TG analysis							
Study population with baseline renal disease (1)	Proportion	0.6321	0.4955	0.3556	0.2743	0.2256	0.1941
	At risk	1769	1156	633	406	249	172
Study population with no baseline renal disease (2)	Proportion	0.6012	0.4677	0.3365	0.2671	0.2201	0.1872
	At risk	12,190	8116	4630	3042	1785	1199
Comparison group with baseline renal disease (3)	Proportion	0.6298	0.4967	0.3722	0.2945	0.2411	0.2118
	At risk	1731	1170	673	456	278	190
Comparison group with no baseline renal disease (4)	Proportion	0.5938	0.4689	0.3404	0.2724	0.2247	0.1911
	At risk	12,065	8226	4741	3181	1840	1245
Clustered *P* values for between-group comparisons in the elevated-TG analysis							
		1 vs 2	1 vs 3	1 vs 4	2 vs 3	2 vs 4	3 vs 4
		0.054	0.252	0.070	<0.001	0.775	0.001
B) High-TG analysis							
Study population with baseline renal disease (1)	Proportion	0.6332	0.4797	0.3384	0.2602	0.2041	0.1741
	At risk	824	511	268	175	102	70
Study population with no baseline renal disease (2)	Proportion	0.5853	0.4533	0.3216	0.2535	0.2043	0.1736
	At risk	5641	3730	2103	1370	797	535
Comparison group with baseline renal disease (3)	Proportion	0.6305	0.4963	0.3637	0.2795	0.2374	0.2118
	At risk	819	554	319	210	135	92
Comparison group with no baseline renal disease (4)	Proportion	0.5814	0.4547	0.3288	0.2656	0.2213	0.1899
	At risk	5609	3774	2155	1447	838	558
Clustered *P* values for between-group comparisons in the high-TG analysis							
		1 vs 2	1 vs 3	1 vs 4	2 vs 3	2 vs 4	3 vs 4
		0.130	0.118	0.309	<0.001	0.271	0.003

Kaplan-Meier analysis. Clustered *P* values were calculated using Cox proportional hazard model with cohort as independent variable. Study population is the elevated-TG cohort (TG ≥150 mg/dL) and high-TG subcohort and their propensity score matched comparators with TG < 150 mg/dL and HDL-C > 40 mg/dL

*HDL-C* high-density lipoprotein cholesterol, *TG* triglycerides

### Subgroup analyses of statin persistence

**Fig. 2:**
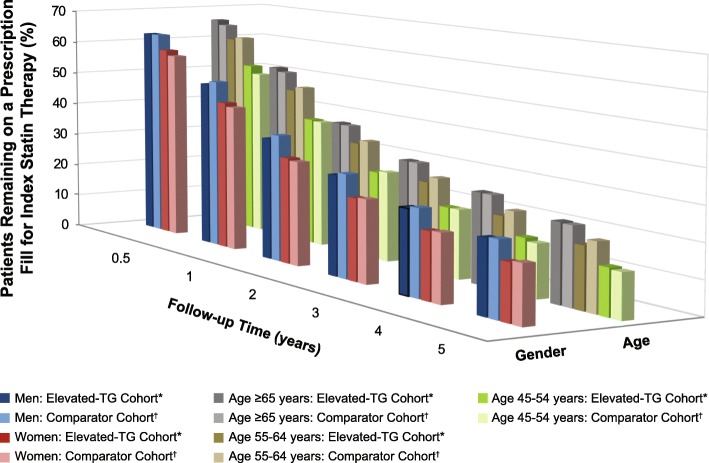
Persistence to Index Statin Therapy by Patients With High CV Risk According to TG Level, Gender, and Age. Kaplan-Meier analysis. Clustered *P* values were calculated using cohort and gender. See Tables 4 and 5 for *P* values. *P* < 0.001 for comparisons between men vs women and younger vs older patients. *TG ≥150 mg/dL. ^†^TG < 150 mg/dL and HDL-C > 40 mg/dL; propensity score matched to elevated-TG cohort. CV, cardiovascular; HDL-C, high-density lipoprotein cholesterol; TG, triglycerides

Across all subgroups, using Kaplan-Meier analysis, the probability of remaining on a prescription fill for index statin was < 54% at 1 year and < 25% at 5 years. Statin persistence was worse in women than in men (*P* < 0.001; Fig. 2 and Table 4). In the elevated-TG cohort, the probability of remaining on a prescription fill for index statin therapy after 5 years was 21.3% for men and 16.3% for women, compared with 21.7 and 16.9% in the comparator cohort, respectively. Statin persistence was worse in younger than in older patients (*P* < 0.001; Table 5). In the elevated-TG and comparator cohorts, respectively, the probability of remaining on a prescription fill for index statin therapy after 5 years was 14 and 13% for patients aged 45–54 years, 18 and 20% for patients aged 55–64 years, and 23% in both cohorts for patients aged ≥ 65 years.

**Fig. 3:**
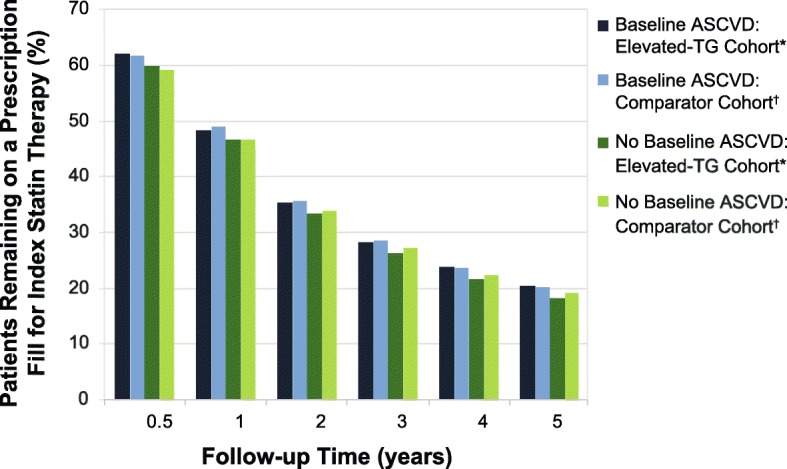
Persistence With Index Statin in High CV Risk Patients by TG Level and Baseline ASCVD. Clustered *P* values were calculated using cohort and baseline ASCVD. See Table 6 for *P* values. *P* < 0.01 for comparisons between patients with and without baseline ASCVD. *TG ≥150 mg/dL. ^†^TG < 150 mg/dL and HDL-C > 40 mg/dL; propensity score matched to elevated-TG cohort. ASCVD, atherosclerotic cardiovascular disease; HDL-C, high-density lipoprotein cholesterol; TG, triglycerides

By contrast, in patients with baseline ASCVD, the probability of remaining on a prescription fill for index statin therapy after 5 years was slightly higher than in those without baseline ASCVD, although persistence was still poor (*P* = 0.001 and *P* = 0.002 for the elevated-TG cohort and the comparator cohort, respectively: Fig. 3; Table 6). In the elevated-TG cohort, the probability of remaining on a prescription fill for index statin therapy after 5 years was 20.3% for patients with baseline ASCVD, compared with 18.2% for those without baseline ASCVD; these probabilities were 20.2 and 19.0% for those with and without baseline ASCVD in the comparator cohort, respectively (Table 6).

Patients without diabetes at baseline had a higher probability of remaining on a prescription fill for index statin over the course of the study than those with diabetes (*P* = 0.029 and *P* = 0.002 for the elevated-TG cohort and comparator cohort, respectively) but again, persistence was low (Table 7). In the elevated-TG cohort, the probability of remaining on a prescription fill for index statin therapy after 5 years was 20.8% for patients without baseline diabetes and 18.4% for those with baseline diabetes; similarly, the probability of remaining on a prescription fill for index statin therapy was 19.0 and 21.0% for those with and without baseline diabetes, respectively, in the comparator cohort (Table 5). There were no significant differences in persistence among patients with or without diabetes between the elevated-TG and comparator cohorts.

Similar trends of poor persistence were seen in other subgroups, with patients with a history of peripheral arterial revascularization, heart failure, PAD, and renal disease at baseline all having greater probability of persistence (although still poor) than those with no history (comparisons did not reach statistical significance; Tables 8, 9, 10 and 11). There was also no difference in statin persistence between those with elevated TG and propensity-matched comparators in these subgroups.

## Discussion

Results from this 5-year retrospective administrative claims analysis indicate that persistence with index statin therapy is poor in patients with elevated TG (≥ 150 mg/dL) or high TG (200–499 mg/dL), diabetes, and/or ASCVD. This result is consistent with a number of previous studies over the past two decades, and confirms that poor long-term statin persistence remains an issue of concern for patients with high CV disease risk, including those with elevated TG who may be at increased risk of CV events and patients with diabetes [4–6, 8, 10, 16, 17]. This highlights the fact that very little, if anything, has changed in the last two decades with regard to improving statin persistence, which remains abysmal in all groups probed in our study, all of whom are at high risk of CV events. This low persistence has a significant effect on risk for CV events, including death. A systematic review found that statin discontinuation was associated with an increased risk of death or CV events [17]. In a Danish population study of nearly 675,000 individuals, early statin discontinuation increased with negative reports about statins in the news media and was associated with increased risk of myocardial infarction and death from CV disease, whereas early statin discontinuation decreased with positive news media reports about statin [18]. In a population study from the United Kingdom, statin discontinuation after acute myocardial infarction was associated with higher total mortality than any other pattern of statin prescription [19]. A recent analysis in a Veterans Affairs population found that poor statin adherence, particularly high-intensity statins, was associated with a higher incidence of all-cause mortality [7]. Another study estimated that improving adherence from 50 to 75% could double the number of deaths prevented [20].

Given the consistently low adherence to statin therapy in all of these studies, it is important to consider what steps could be taken to remedy this issue. A number of modifiable factors associated with patient out-of-pocket costs, including use of generic versus brand-name statins, low or no copayments, and coupons, have been identified [21]. Statin intolerance due to adverse effects may be another important reason for discontinuing therapy [22]; in an internet survey of statin users, the primary reason for stopping statin therapy was side effects (primarily muscle-related side effects) in 62% [23]. Poor statin adherence due to intolerance has been associated with an increased risk of recurrent myocardial infarction and coronary heart disease events [24]. Providing support for and careful assessment of patients who report side effects that are potentially related to statins may help improve adherence and persistence [25]. Physicians should address any side-effect–related concerns that patients have, and, if necessary, titrate the dose or switch to another statin [26]. Alternate-day dosing of statins is another option for patients with statin intolerance [27]. Cholesterol management guidelines recommend proactively screening for muscle issues prior to initiating and during statin therapy, including measuring creatine kinase levels in those at greatest risk, in order to proactively manage this side effect and distinguish from unrelated muscle issues to ensure continued persistence [1].

Another possible reason for the low persistence in the population described here is the burden of polypharmacy [28]. Approximately 85% of patients in this study had diabetes, 79% had hypertension, and 29% had a history of ASCVD, in addition to other comorbidities. Most patients were therefore likely taking several medications multiple times a day, leading to reduced persistence [28, 29]. One retrospective study in a Veterans Health Administration population suggested that statin adherence actually correlated with the number of drugs that patients were taking at baseline [30]. Other factors that may affect adherence and persistence include illness; beliefs about the intervention in question and its perceived risks, benefits, and necessity; patient–practitioner relationship; physical and mental illness; and financial constraints [29, 31]. The high rate of diabetes, hypertension, and other comorbidities in this population, and the resulting polypharmacy, suggest that these persistence data are not generalizable to patients with simple hypertriglyceridemia.

Statin nonadherence and nonpersistence have been associated with younger patient age, female gender, lower income, and nonwhite race [31–33]. This is in agreement with the results of this study, which found that female gender and younger age were associated with significantly lower statin persistence over 5 years of follow-up. Of note, while previous studies have suggested that concomitant diabetes is predictive of better adherence and persistence, our study found slightly lower persistence in patients with diabetes [32]. This may reflect the study design, which required that all patients have diabetes or ASCVD. As a result, all patients without diabetes had ASCVD, which may be associated with a higher rate of statin persistence than diabetes. Persistence with medications for asymptomatic diseases, such as hypercholesterolemia, is also a challenge because of the lack of noticeable efficacy by the patient in everyday life; this may explain in part the low persistence with statin therapy observed here [31]. Regardless of the reasons patients are not continuing their index statin, this study emphasizes that statin persistence is alarmingly poor and is likely contributing to adverse health outcomes in these high-risk patients.

This study has a number of limitations. Data used in the study were collected for administration of health claims, not for research. The included population was limited to patients in a managed care health plan in the United States and may not be generalizable to other populations. In addition, medication usage claims do not indicate whether the medication was consumed or whether it was taken as prescribed. The data may also contain inaccurate recording of health events, missing data, and uncertainty about internal validity [34, 35]. Laboratory test results, including lipid measures during the follow-up period, were only available for a subset of patients, but the extent of missing data may not be distinguishable from the lack of an administered test. The analysis measured only persistence with statin index therapy as a class and did not measure whether patients who discontinued index statin resumed therapy with another lipid-lowering medication class. This analysis was designed to assess health status and burden over time in patients with elevated TG despite having generally controlled LDL-C, and was not designed to assess the potential effects of any treatment modality. Statistical analyses should be evaluated in the context of the large sample size; this may indicate statistical significance for some parameters even when differences are small and not clinically meaningful. This is potentially enhanced by the large number of statistical comparisons conducted across various subgroups which may have introduced type 1 errors. Despite these limitations, real-world data are pragmatic in that they examine patient populations in the context of clinical practice and may be more reflective of actual use in practice than evidence from clinical trials [34, 36].

## Conclusions

This study highlights that persistence with statin therapy is very poor. Although most patients at increased CV risk—including those with ASCVD, elevated or high TG, heart failure, PAD, renal disease, and a history of revascularization—had slightly better probability of persistence than those who did not, persistence remained low after 5 years. These findings underscore the need to develop public health programs and nationwide patient education initiatives about the well-defined benefits of statin therapy, particularly in the high-risk setting. The crucial need to ensure long-term statin persistence in high-risk patients should also be reinforced at all patient follow-up visits. Helping patients understand that statin discontinuation correlates with increased risk for acute CV events and death is a matter that cannot be overemphasized. Institution of programs to enhance persistence and adherence to statin therapy, especially in women and younger patients, is also required.

## Data Availability

Data are proprietary to Optum and cannot be shared.

## Abbreviations

ASCVD: Atherosclerotic cardiovascular disease
CV: Cardiovascular
HDL-C: High-density lipoprotein cholesterol
LDL-C: Low-density lipoprotein cholesterol
PAD: Peripheral artery disease
TG: Triglycerides
